# Evaluation of a point-of-care calcium device in bovine plasma and serum

**DOI:** 10.3168/jdsc.2022-0346

**Published:** 2023-07-13

**Authors:** F.A. Leal Yepes, E. Behling-Kelly, L.S. Caixeta, L. Tikofsky, L. Parrish, K.N. Heaton

**Affiliations:** a Department of Veterinary Clinical Sciences, College of Veterinary Medicine, Washington State University, Pullman 99164-6610; b Department of Population Medicine and Diagnostic Sciences, Cornell University, Ithaca, NY 14853; c Department of Veterinary Population Medicine, University of Minnesota, St. Paul 55108; d Boehringer Ingelheim Animal Health USA Inc., Duluth, GA 30096

## Abstract

Hypocalcemia is a common metabolic disease in dairy cows, and it is defined as total calcium (tCa) blood concentration <2.0 mmol/L. The alternatives for the gold standard test to measure tCa in bovine blood are limited. Therefore, our objective was to compare the performance of the calcium (Ca) point-of-care compact analyzer (POC; ARKRAY Inc.) device with the gold standard method to measure bovine blood tCa concentration. Blood samples (n = 151) from dairy cows were collected within 24 h postpartum from multiparous and primiparous dairy cows for serum and plasma. Then, serum and plasma were stored at −80°C until further analyses with the gold standard method on an automatic analyzer (Cobas C501 analyzer; Roche Diagnostics) and the POC device. The tCa blood concentration was measured in the laboratory in plasma and serum samples using both methods within 10 mo of sample collection. Correlation coefficients (Spearman), coefficients of variation (CV, %), sensitivity, specificity, positive predictive value (PPV), negative predictive value (NPV), accuracy, Passing and Bablok regression, and Bland-Altman agreement test were performed between the gold standard and the POC device. The range and median tCa plasma concentrations measured with the POC device were 1.1 to 2.8 mmol/L and 2.4 mmol/L, respectively. The range and median tCa serum concentrations measured with the POC device were 1.1 to 2.7 mmol/L and 2.3 mmol/L, respectively. The tCa blood concentrations range and median with the gold standard were 1.1 to 2.6 mmol/L and 2.3 mmol/L. The hypocalcemia prevalence of our study population was 11.2%. The CV were 1.89% and 0.55% for low and high tCa in plasma samples measured with the POC, respectively. The CV were 2.57% and 1.58% for low and high tCa in serum, respectively. The Spearman correlation coefficient showed a strong correlation between the gold standard and the POC device for both serum and plasma tCa concentration. The sensitivity of the POC device for both plasma (41.1%) and serum (64.7%) Ca was poor. However, the specificity of the POC device was perfect in plasma (99.2%) and serum (99.2%). The PPV in plasma and serum were 87.5% and 91.6%, respectively. Negative predicted values were 93.0% and 95.6% in plasma and serum. The mean (95% CI) difference between the gold standard and the POC device in plasma and serum were 0.35 (−0.52, 1.23) mmol/L and 0.19 (−0.53, 0.92) mmol/L, respectively. Finally, we observed a strong correlation between the POC device and the gold standard method for tCa plasma and serum. However, the clinical application of the POC device should be carefully considered because its ability to detect cows with hypocalcemia in serum or plasma samples was poor. However, the device performed better than previously analyzed POC devices and needs further improvement to be a valuable tool for the dairy industry.

## Highlights

• The correlation between the POC device and gold standard was strong in serum and plasma.
• The sensitivity of the POC device was poor in serum and plasma.
• The specificity of the POC device was excellent in serum and plasma.

## Graphical Abstract

**Figure:**
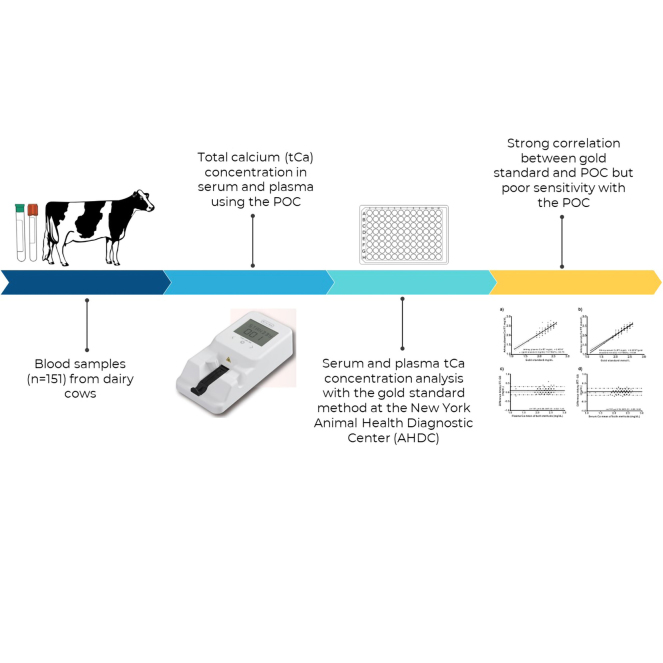
**Summary:** Serum and plasma from dairy cows were collected to measure total calcium (Ca) concentration using the Ca point-of-care compact analyzer (POC; ARKRAY Inc.) device and a gold standard method. The performance of the POC analyzer was estimated using correlation coefficients (Spearman), coefficient of variation (%), sensitivity, specificity, Passing and Bablok regression, and Bland-Altman agreement test. We found a strong correlation between the POC device and the gold standard method for total Ca plasma and serum. However, the clinical application of the POC device should be carefully considered because its ability to detect cows with hypocalcemia in serum or plasma samples was poor.

Hypocalcemia is defined as low blood calcium (Ca) blood concentration <2.0 mmol/L (Reinhardt et al., 2011). The first 24 h postpartum was commonly the most critical period for hypocalcemia in dairy cows. However, recent work has shown that cows with persistent hypocalcemia during the first 4 DIM are at greater risk of developing adverse outcomes (McArt and Neves, 2020; Seely et al., 2021; Seely and McArt, 2022). Yet, the association of hypocalcemia and fresh cow disorders has been recently challenged, as immune activation may lead to hypocalcemia and might be the underlying cause for hypocalcemia at 4 DIM (Horst et al., 2021).

Moreover, hypocalcemia is also one of most common metabolic disorders in dairy cows. Its negative consequences on cows make hypocalcemia one of the main welfare concerns of dairy farmers and veterinarians. The USDA (2018) reported that 77.2% of all dairy operations in the United States were affected by hypocalcemia. Similarly, 47.6% of multiparous cows in German dairy herds suffer from hypocalcemia within 48 h after parturition (Venjakob et al., 2021). Hypocalcemia is a common issue in both intensive-confined and pasture-based dairy production systems, producing an important burden for dairy farms (Roche, 2003; Oetzel, 2013). In addition, hypocalcemia has been associated with the development of other diseases in dairy cows, such as displaced abomasum and metritis, and decreased reproductive performance (Venjakob et al., 2017; Neves et al., 2018a). However, hypocalcemia is rarely prevalent in primiparous cows (Reinhardt et al., 2011; Venjakob et al., 2017).

Herd-based monitoring of hypocalcemia is rarely performed because the assay to estimate blood calcium concentration is time sensitive, and the optimal timing for sampling is variable (Neves et al., 2018a). Total calcium (**tCa**) is the more stable Ca form in bovine blood and is the most commonly measured by diagnostic laboratories, but tCa includes components that may not be readily biologically available because it is bound to other blood elements (Oetzel, 2004).

The gold standard tests for the determination of tCa concentrations in either plasma or serum are costly and, most of the time, difficult to access for veterinarians and dairy farmers. While there is an alternative to the laboratory-based gold standard test, the iSTAT that can measure ionized Ca, the equipment and test are costly (equipment ≥$8,500 and $18/sample) and thus impractical for the vast majority of dairy farmers and field veterinarians. The VetStat Electrolyte Blood Gas Analyzer (IDEXX Laboratories Inc.) is another alternative but the equipment is costly and provides additional electrolyte measurements that may not be relevant for dairy producers or practitioners. Therefore, a lower-cost, point-of-care (**POC**) device, with accuracy and precision comparable to the gold standard, will provide dairy farmers with access to routine measurement of blood calcium concentration in dairy cows. Therefore, our objective was to compare the performance of a tCa POC device with the gold standard method to measure bovine blood tCa concentration in serum and plasma samples.

Blood samples (n = 151) from dairy cows were collected at one commercial farm in Washington state between February 2020 and July 2021. All of the animal procedures were approved by the Institutional Animal Care and Use Committee (IACUC) at Washington State University (protocol #6855). The sample size was calculated based on a 50% incidence of hypocalcemia in the US dairy cow population (Neves et al., 2018a) and an expected incidence of 38% of hypocalcemia in our study population with a confidence level of 95% and power of 90%. The sample size calculation was performed using the web application ClinCalc (Kane, 2022). All blood samples were collected within 24 h postpartum from dairy cows (first lactation n = 69; second lactation n = 46; third lactation n = 21; and fourth or higher lactation n = 15). Blood was collected from the coccygeal vessels using 10-mL blood collection tubes (Becton Dickinson) containing 158 USP (United States Pharmacopeia) of lithium heparin and a 10-mL tube without anticoagulant, using 20-gauge × 2.54-cm blood collection needles. The samples were placed on ice immediately after collection and then centrifuged at 3,000 × *g* at room temperature for 20 min for serum and plasma separation. Serum and plasma were stored at −80°C until further analysis with the gold standard method on an automatic analyzer (Cobas C501 analyzer; Roche Diagnostics) at the New York Animal Health Diagnostic Center and the POC device. The tCa blood concentration was measured using the Ca POC compact analyzer (ARKRAY Inc.) in the laboratory using both plasma and serum samples at room temperature, following the manufacturer's instructions. Briefly, start the POC device and wait around 10 min for the machine to be warmed up and ready. It will show “STANDBY” on the display when it is ready. Calibrate the device using the chip contained in each box of test strips. Then, load the test strip onto the strip tray, with the wells toward the device, and using the pipette provided with the devices and a universal 10-µL pipette tip, transfer 4 µL of plasma or serum into the well of the test strip. The measurements take approximately 2 min after pressing the round button on the device and inserting the test strip. All samples were analyzed with the gold standard and the POC within 10 mo of sample collection. The tCa concentration in plasma and serum is stable for up to 12 mo when stored at −80°C (Bach et al., 2020).

Data analysis was performed in SAS (SAS 9.4, SAS Institute Inc.). Correlation coefficients (Spearman) between tCa gold standard and the POC device were computed using Proc Corr (SAS 9.4). Coefficients of variation (%) were determined for plasma and serum samples, each in high (2.6 mmol/L) and low (1.1 mmol/L) concentrations of tCa by measuring each sample 4 times using the POC device. The sensitivity of the method in both plasma and serum was calculated as the proportion of animals properly diagnosed as positive for hypocalcemia (tCa blood concentration <2.0 mmol/L; Reinhardt et al., 2011) among all animals identified as positive by the POC device compared with those classified as positive by the gold standard. Specificity was calculated as the proportion of animals diagnosed adequately as negative for hypocalcemia among all animals identified as negative by the POC device compared with those classified as negative by the gold standard in plasma and serum. The 95% confidence intervals (95% CI) were calculated using Proc FREQ in SAS for sensitivity and specificity values. The positive predictive value (**PPV**) was calculated as the probability that the disease is present when the test is positive. The negative predictive value (**NPV**) was estimated as the probability that the disease is not present when the test is negative. The PPV and NPV were calculated using MedCalc Statistical Software (v 20.106, MedCalc Software Ltd.; https://www.medcalc.org; 2022). The test accuracy was calculated as the overall probability that a patient is correctly classified in MedCalc Statistical Software. Regression analyses between the gold standard tCa concentrations and the POC results were performed using Proc REG in SAS. Linearity between methods was graphically assessed using the plot of residuals. Passing and Bablok regression coefficients, slopes, and intercepts were obtained using MedCalc Statistical Software. Bland-Altman plots and the 95% CI of the agreement were generated with GraphPad Prism (v. 9.3.1).

The range and median tCa plasma concentrations measured with the POC device were 1.1 to 2.8 mmol/L and 2.4 mmol/L, respectively. The range and median tCa serum concentrations measured with the POC device were 1.1 to 2.7 mmol/L and 2.3 mmol/L, respectively. The range and median tCa blood concentrations with the gold standard were 1.1 to 2.6 mmol/L and 2.3 mmol/L, respectively. The coefficients of variation were 1.9% and 0.6% in plasma samples with high or low tCa measured with the POC device, respectively. The coefficients of variation were 2.6% and 1.6% in serum samples with low and high tCa concentration measured with the POC device, respectively. The hypocalcemia prevalence, tCa blood concentration <2.0 mmol/L, in our study population was 11.2%. The Spearman correlation coefficient showed a strong correlation between tCa serum and the gold standard (r = 0.84, *P* ≤ 0.001). Similarly, the Spearman correlation was strong between tCa plasma concentration with the POC device and the gold standard (r = 0.81, *P* ≤ 0.001). In our study, the main objective was to compare the performance of a tCa POC device with the gold standard method to measure bovine blood tCa. The tCa concentration correlation between gold standard and the POC device is comparable to previous validations of different Ca POC (Peiró et al., 2010; Couto Serrenho et al., 2021; Suzuki et al., 2021). The sensitivity, specificity, PPV, and NPV of the POC device in plasma and serum are summarized in Table 1. The sensitivity of the POC device for both plasma and serum Ca was poor. The specificity of the POC device was perfect in plasma and serum. In the conditions of our study, the Ca POC compact analyzer did not perform well in differentiating cows with hypocalcemia (tCa <2.0 mmol/L). The accuracy (95% CI) of the POC device was 95.3% (90.6, 98.1) and 92.7% (87.3, 96.3) in serum and plasma, respectively. The PPV, NP, and accuracy of the test were greater in the current study compared with a similar device recently analyzed (Couto Serrenho et al., 2021).

**Table 1:** Performance of a point-of-care (POC) test device for the classification of hypocalcemia, defined as total Ca (tCa) blood concentration <2.0 mmol/L

Test^1^	Sensitivity^2^ (%; 95% CI)	Specificity^3^ (%; 95% CI)	PPV^4^ (%; 95% CI)	NPV^5^ (%; 95% CI)
Plasma tCa POC compact analyzer	41.1 (17.7, 64.5)	99.2 (97.8, 100.0)	86.7 (0.6, 99.9)	92.9 (87.7, 96.1)
Serum tCa POC compact analyzer	64.7 (41.9, 87.4)	99.2 (97.8, 100)	91.2 (1.3, 99.9)	95.6 (88.2, 98.4)

1 The tCa POC compact analyzer (ARKRAY Inc.).

2 Sensitivity was calculated as the proportion of animals that were classified as positive for hypocalcemia by the meter compared with the animals classified as positive with the gold standard method (tCa blood concentration <2.0 mmol/L) performed at the New York Animal Health Diagnostic Center (AHDC, Ithaca, NY).

3 Specificity was calculated as the proportion of animals that were classified as negative for hypocalcemia by the meter as compared with the number of animals classified as negative by the gold standard method (tCa blood concentration ≥2.0 mmol/L) performed at the New York AHDC.

4 The positive predictive value (PPV) was calculated as the probability that the disease is present when the test is positive.

5 The negative predictive value (NPV) was estimated as the probability that the disease is not present when the test is negative.

Linear regressions between the gold standard and POC device are presented in Figure 1. Passing and Bablok is a nonparametric regression method, and the results of the analysis are presented in Table 2. Similar to the Spearman, we observed a strong correlation between the gold standard method and the POC device in plasma and serum using the Passing and Bablok regression. Test agreement among gold standard tCa and the POC device is presented in Figures 1C and 1D. The mean (95% CI) difference between the gold standard and POC device in plasma and serum were 0.35 (−0.52, 1.23) mmol/L and 0.19 (−0.53, 0.92) mmol/L, respectively. Our results suggested that, on average, the POC device overestimates the tCa blood concentration. Our results are similar to previous studies intending validation of POC Ca in whole blood, plasma, and serum. Unfortunately, the precision and accuracy of these POC devices to measure blood Ca for field or laboratory use are inconsistent (Neves et al., 2018b; Couto Serrenho et al., 2021).

**Figure 1:**
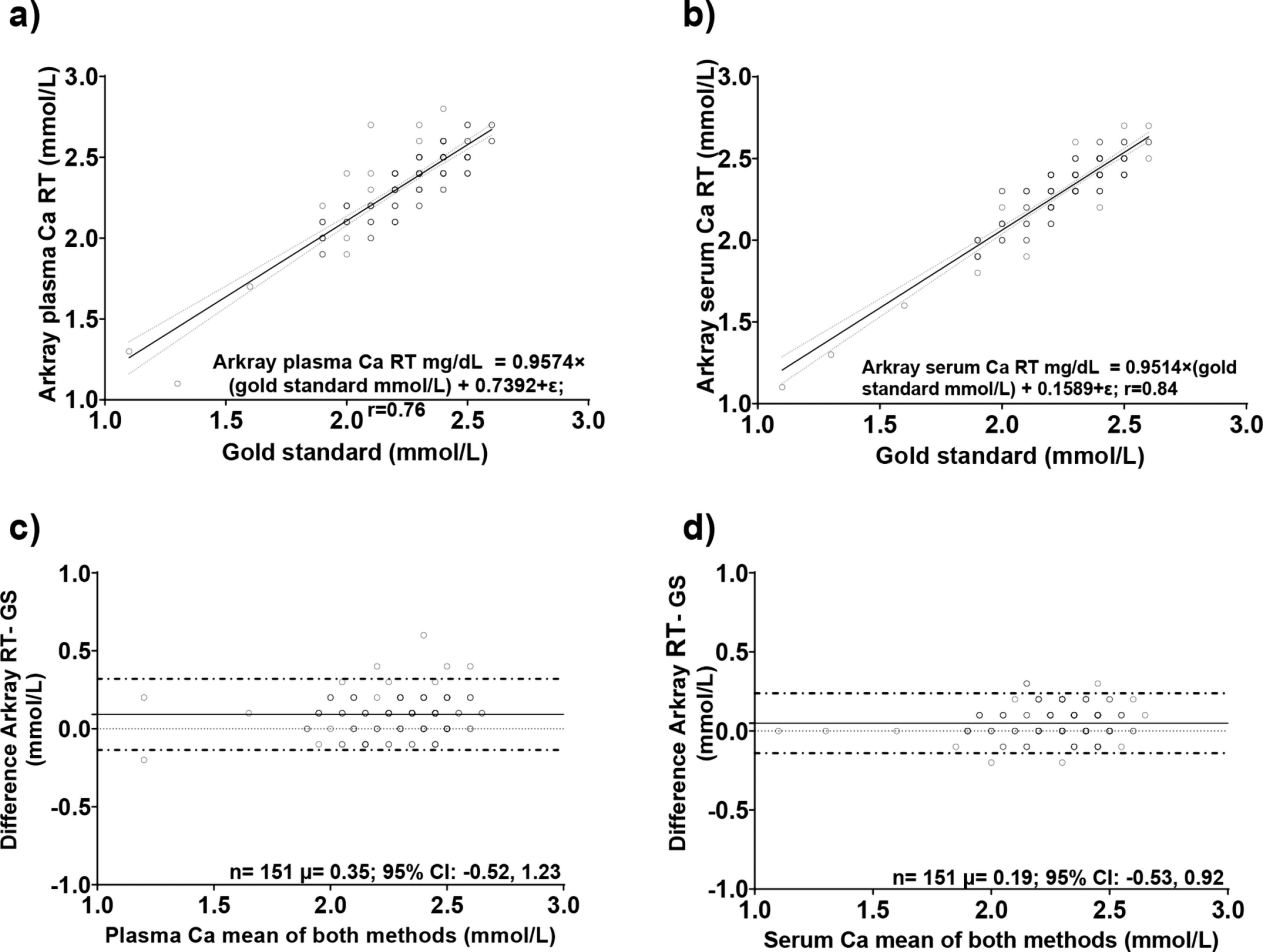
Linear regression analysis between total calcium (tCa; mmol/L) blood concentration measured with the gold standard method at the New York Animal Health Diagnostic Center and (a) tCa plasma concentration with the tCa point-of-care compact analyzer (ARKRAY Inc.); (b) tCa serum concentration with the Ca point-of-care compact analyzer (ARKRAY Inc.). Bland-Altman plots of the difference in tCa concentrations for (c) plasma tCa point-of-care compact analyzer versus tCa gold standard and (d) serum tCa concentration point-of-care compact analyzer versus tCa gold standard. The solid horizontal line represents the mean bias; horizontal dashed lines represent the 95% confidence interval of agreement. µ is the overall mean bias (mmol/L) calculated as the Ca point-of-care compact analyzer gold standard. A positive mean bias shows an overestimation of the tCa concentration by the point-of-care analyzer compared with the gold standard. RT = room temperature; GS = gold standard method; ε = error, which represents the combined effect of omitted variables and is independent across subjects.

**Table 2:** Passing and Bablok regression coefficients for total calcium (tCA) concentrations in whole blood with a point-of-care device

Test^1^		Gold standard^2^	Intercept	Slope	Correlation coefficient (95% CI)	*P*-value
Plasma tCa		tCa	0.1	1.0	0.81 (0.75, 0.86)	<0.0001
Serum tCa		tCa	0.0	1.0	0.83 (0.78, 0.88)	<0.0001

1 tCa (mmol/L) point-of-care compact analyzer (ARKRAY Inc.).

2 The tCa (mmol/L) blood concentration gold standard test was performed at the New York Animal Health Diagnostic Center (Ithaca, NY).

The disease prevalence is relatively low in the current study. The PPV and NPV may be affected by low disease prevalence in the current study. The PPV and NPV are difficult to transfer to scenarios with different prevalence levels because PPV and NPV depend on disease prevalence. Conversely, sensitivity and specificity may be less affected by varying levels of disease prevalence (Leeflang et al., 2013).

Finally, our study showed a strong correlation, great accuracy, and agreement between the tCa POC device and the gold standard in plasma and serum. However, we recommend caution when using this POC device due to its poor sensitivity. Further modifications of the POC device are needed to increase its ability to identify dairy cows with hypocalcemia.
